# The impact of gene polymorphism and hepatic insufficiency on voriconazole dose adjustment in invasive fungal infection individuals

**DOI:** 10.3389/fgene.2023.1242711

**Published:** 2023-08-24

**Authors:** Guolin Li, Qinhui Li, Changji Zhang, Qin Yu, Qi Li, Xiaoshi Zhou, Rou Yang, Xuerong Yang, Hailin Liu, Yong Yang

**Affiliations:** ^1^ Department of Pharmacy, Sichuan Academy of Medical Sciences and Sichuan Provincial People’s Hospital, School of Medicine, University of Electronic Science and Technology of China, Chengdu, China; ^2^ School of Basic Medicine and Clinical Pharmacy, China Pharmaceutical University, Nanjing, China; ^3^ Department of Medical, Sichuan Academy of Medical Sciences and Sichuan Provincial People’s Hospital, School of Medicine, University of Electronic Science and Technology of China, Chengdu, China; ^4^ College of Pharmacy, Southwest Medical University, Luzhou, China; ^5^ Personalized Drug Therapy Key Laboratory of Sichuan Province, School of Medicine, University of Electronic Science and Technology of China, Chengdu, China; ^6^ Department of Pharmacy, The People’s Hospital of Chongqing Liangjiang New Area, Chongqing, China

**Keywords:** voriconazole, genetic polymorphism, hepatic insufficiency, CYP2C19, invasive fungal infections, dose adjustment

## Abstract

Voriconazole (VRZ) is a broad-spectrum antifungal medication widely used to treat invasive fungal infections (IFI). The administration dosage and blood concentration of VRZ are influenced by various factors, posing challenges for standardization and individualization of dose adjustments. On the one hand, VRZ is primarily metabolized by the liver, predominantly mediated by the cytochrome P450 (CYP) 2C19 enzyme. The genetic polymorphism of CYP2C19 significantly impacts the blood concentration of VRZ, particularly the trough concentration (Ctrough), thereby influencing the drug’s efficacy and potentially causing adverse drug reactions (ADRs). Recent research has demonstrated that pharmacogenomics-based VRZ dose adjustments offer more accurate and individualized treatment strategies for individuals with hepatic insufficiency, with the possibility to enhance therapeutic outcomes and reduce ADRs. On the other hand, the security, pharmacokinetics, and dosing of VRZ in individuals with hepatic insufficiency remain unclear, making it challenging to attain optimal Ctrough in individuals with both hepatic insufficiency and IFI, resulting in suboptimal drug efficacy and severe ADRs. Therefore, when using VRZ to treat IFI, drug dosage adjustment based on individuals’ genotypes and hepatic function is necessary. This review summarizes the research progress on the impact of genetic polymorphisms and hepatic insufficiency on VRZ dosage in IFI individuals, compares current international guidelines, elucidates the current application status of VRZ in individuals with hepatic insufficiency, and discusses the influence of CYP2C19, CYP3A4, CYP2C9, and ABCB1 genetic polymorphisms on VRZ dose adjustments and Ctrough at the pharmacogenomic level. Additionally, a comprehensive summary and analysis of existing studies’ recommendations on VRZ dose adjustments based on CYP2C19 genetic polymorphisms and hepatic insufficiency are provided, offering a more comprehensive reference for dose selection and adjustments of VRZ in this patient population.

## 1 Introduction

Invasive fungal infection (IFI) is a dangerous disease commonly seen in individuals with damaged immune function, such as individuals with acquired immune deficiency syndrome (AIDS), malignancy, and organ transplantation (Kullberg and Arendrup, 2015; Douglas et al., 2021). Individuals with hepatic insufficiency are vulnerable to IFI due to their low immune function and increased intestinal mucosal permeability, and IFI has a high mortality rate, which seriously affects patient prognosis, especially in immunosuppressed individuals, where the mortality rate may reach up to 90% (Yamada et al., 2018; Chen et al., 2021; Jenks et al., 2021). VRZ is a medication with broad-ranging antifungal properties extensively used to treat IFI (Ullmann et al., 2007). However, the pharmacokinetic parameters, efficacy, and safety of VRZ are influenced by various factors, such as genetic polymorphisms, liver function, and drug interactions (Liu and Mould, 2014; Lamoureux et al., 2016).

Gene polymorphism is one of the critical factors in the variability of pharmacokinetic parameters of VRZ. Gene polymorphism is when multiple versions of genes are present in a population, and these versions can result in different enzyme activities (Cheng et al., 2018). VRZ is primarily metabolized in the liver by CYP2C19 enzyme and partially by CYP3C4 and CYP2C9. CYP2C19 gene polymorphism affects the pharmacokinetic parameters and efficacy of VRZ (Hulin et al., 2011; Hamadeh et al., 2017). It has been found that the pharmacokinetic parameters of VRZ in mutant carriers such as CYP2C19*2 are significantly higher than those in wild-type carriers, while enhanced carriers such as CYP2C19*17 show the opposite trend (Pascual et al., 2008; Dolton et al., 2012). Therefore, individualized dose adjustment strategies are needed for different CYP2C19 genotypes to improve the efficacy and safety of VRZ (Pascual et al., 2012).

Hepatic insufficiency can also affect the pharmacokinetic parameters and efficacy of VRZ. Individuals with hepatic insufficiency experience difficulty breaking down and eliminating drugs from their system, and this causes the drug to remain in the body for an extended duration, leading to higher concentrations of the drug (Liu and Mould, 2014). Therefore, individuals with hepatic insufficiency should decrease their VRZ dosage to prevent ADRs caused by a potential overdose (Lamoureux et al., 2016). According to a study, the way VRZ works and its effects differ significantly for individuals with hepatic insufficiency compared to those with normal liver function (Tang et al., 2019; 2021). In addition, VRZ has a small margin of safety and has multiple ADRs, including neurotoxicity, hepatotoxicity, and visual impairment (Levine and Chandrasekar, 2016; Lem et al., 2019). Research has confirmed a meaningful connection between Ctrough and both the effectiveness of the treatment and the adverse drug reactions (Yang et al., 2022). As a result, medical professionals frequently suggest therapeutic drug monitoring (TDM) to enhance patient outcomes (Jin et al., 2016; Luong et al., 2016). The instructions provide dosage adjustment recommendations for individuals with mild to moderate liver impairment, utilizing exposure data from real-world usage of VRZ. However, there is a lack of comprehensive data on the safety, pharmacokinetics, and appropriate dosage for patients with severe hepatic insufficiency. It is crucial to develop individualized dose adjustment strategies for these patients to optimize the efficacy and safety of VRZ treatment.

Based on the above studies, the treatment of IFI involves a significant role for VRZ, but its pharmacokinetic parameters, efficacy and safety are influenced by several factors. Individualized dose adjustment strategies can enhance the effectiveness and safety of VRZ, but there are some differences in the results of different studies. Therefore, when developing dose adjustment strategies, the distribution of genetic polymorphisms and hepatic insufficiency in diverse populations should be considered, and the effects of multiple factors should be taken into account. However, there is not enough large-scale clinical research on personalized dosing for VRZ to confirm whether it is a safe and effective approach for guiding clinical practice. This paper focuses on how the CYP2C19 gene and hepatic insufficiency affect VRZ dose adjustment. From a pharmacogenomic perspective, we further investigate the influence of genetic polymorphisms in CYP3A4, CYP2C9, and ABCB1 on Ctrough. This information can help develop personalized treatment plans for VRZ use in individuals with IFI.

## 2 Voriconazole

**FIGURE 1:**
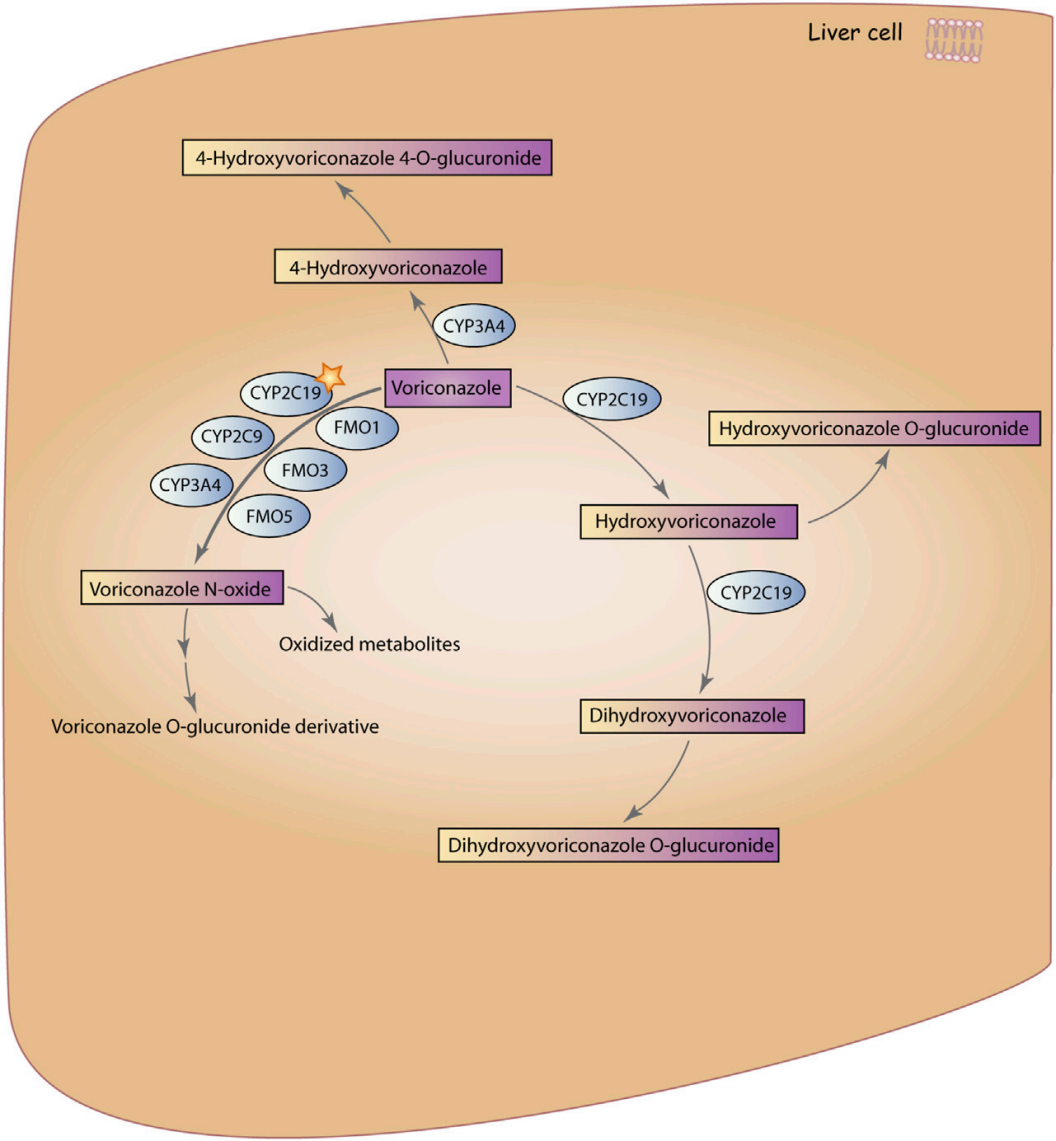
Metabolic pathways of VRZ. VRZ is primarily metabolized by the enzyme CYP2C19 to form Voriconazole N-oxide, with contributions from members of the CYP3A4, CYP2C9, and FMO families. Subsequently, Voriconazole N-oxide is further metabolized to voriconazole O-glucuronidated derivative and other oxidized metabolites. The second metabolic pathway involves hydroxylation of the methyl group of VRZ, in which VRZ is metabolized by CYP3A4 to 4-Hydroxyvoriconazole, which is then glucuronidated to form 4-Hydroxyvoriconazole 4-glucuronidate. The third pathway involves hydroxylation of the fluconazole ring of VRZ, in which VRZ is metabolized by CYP2C19 to hydroxyvoriconazole, which can further undergo hydroxylation by CYP2C19 to form dihydroxyvoriconazole. Finally, dihydroxyvoriconazole is glucuronidated to form dihydroxyvoriconazole O-glucuronidated. Voriconazole Pathway, Pharmacokinetics (Reproduced from PharmaGKB, licensed under CC BY-SA 4.0).

Voriconazole (VRZ) was approved for marketing by the U.S. Food and Drug Administration (FDA) in 2002 and was introduced in China in 2005 (Pallet and Loriot, 2021). VRZ is a synthetic second-generation triazole antifungal drug derived from fluconazole and exhibits broad-spectrum antifungal activity. It is believed that the mechanism of action for triazole antifungal drugs involves inhibiting the fungal enzyme 14α-demethylase, which is responsible for converting lanosterol to ergosterol, thereby disrupting the synthesis of the cell membrane (Thompson and Lewis, 2010). The Infectious Diseases Society of America recommends VRZ as the primary treatment for invasive Aspergillosis (Chen et al., 2018). It is also effective against *Candida* spp. in treatment and prevention (Xing et al., 2017). The metabolic pathways of VRZ are influenced by multiple enzymes, with its primary circulating metabolite being Voriconazole N-oxide (Theuretzbacher et al., 2006; Voriconazole Pathway, Pharmacokinetics, n.d.) (Figure 1).

VRZ is used to treat invasive Aspergillosis, non-neutropenic Candidaemia individuals, and severe invasive infections caused by fluconazole-resistant *Candida*; however, VRZ may cause side effects such as hepatotoxicity, neurotoxicity, photosensitivity, visual disturbances, and osteochondritis with or without hyperfluorosis (Chau et al., 2014). Psychiatric disorders are common ADRs caused by VRZ, characterized by symptoms such as delirium, hallucinations, and emotional excitement (Benitez and Carver, 2019). A study suggested that these symptoms are associated with the distribution and blood concentration of VRZ in the body; during its distribution in the body, VRZ can penetrate the blood-cerebrospinal fluid barrier, resulting in brain tissue concentrations that are 2–3 times higher than plasma concentrations (Ertem et al., 2022). ADRs associated with Ctrough also include hepatotoxicity and other neurological disorders (Hamada et al., 2012; Moriyama et al., 2017). Any form of VRZ can be excreted as a metabolite (98%) and as a prototype (2%) within 48 h of administration, so although it is not uncommon for VRZ to cause psychiatric disorders, psychiatric symptoms can rapidly improve or even disappear in a brief amount of time after discontinuation of the drug (Yi et al., 2017). In individuals with hepatic insufficiency combined with invasive aspergillosis, VRZ remains the drug of choice, but there is still much controversy regarding dose selection (Chen and Ning, 2022).

Reports suggest that VRZ can cause liver injury, but the mechanism of its occurrence is still unclear, and some studies suggest that it is mainly related to VRZ metabolism (Kyriakidis et al., 2017; Zhou et al., 2022). VRZ can be converted to active metabolites in the liver, and such functional products can cause mitochondrial damage directly or through inhibition of CYP proteins, leading to cellular dysfunction or necrosis, thus causing liver injury (Pessayre et al., 2012). There is controversy surrounding the impact of VRZ on liver injury, with some studies suggesting that VRZ-induced liver injury is a dose-dependent ADR, but others suggesting that liver injury does not correlate with drug concentration (Suzuki et al., 2013; Zonios et al., 2014).

The pharmacokinetic profile of VRZ in adults is nonlinear, with a significant increase in Ctrough with increasing medication administration. It is predominantly metabolized oxidatively by cytochrome P450 isoenzymes and secondarily by CYP3A4 and CYP2C9 enzymes, in addition to being a CYP3A4 inhibitor itself, so VRZ has more clinically significant drug interactions (Lee et al., 2022). Some studies have reported that the interaction of VRZ with carbamazepine, efavirenz, ritonavir, rifampin, phenobarbital, rifabutin, and nevirapine affects their pharmacokinetic parameters and efficacy (Chen et al., 2018). Therefore, attention needs to be paid to VRZ interactions with other drugs during treatment. VRZ pharmacokinetics are variable within and between individuals, with influencing factors including age, CYP2C19 gene polymorphisms, hepatic function status, drug interactions, and ingestion, further adding to the concern of clinical response variability (Job et al., 2016; Li et al., 2017; You et al., 2018). In addition, Ctrough can significantly impact clinical response, and routine TDM of VRZ is recommended because of inter-patient variability in VRZ pharmacokinetics to avoid high Ctrough-related toxicity and treatment failure with low Ctrough (Denning et al., 2002).

## 3 Hepatic insufficiency combined with IFI

Hepatic insufficiency refers to the damage of liver cells by various hepatogenic factors, resulting in dysfunction of synthesis, degradation, detoxification, storage, secretion, and immunity, which may lead to jaundice, hemorrhage, infection, renal dysfunction, and hepatic encephalopathy (Verma et al., 2022). The advanced stage of hepatic insufficiency is generally called hepatic failure, and the main clinical manifestations are hepatic encephalopathy and hepatorenal syndrome (Rose et al., 2020; Vasques et al., 2022). Individuals with hepatic insufficiency are prone to complications of various infections due to impaired immune function, dysbiosis of intestinal flora, and reduced number of hepatic Kupffer cells, resulting in low immunity (Yamada et al., 2018). Moreover, the application of broad-spectrum, potent antimicrobial drugs and glucocorticoids, as well as invasive procedures, greatly raises the risk of opportunistic infections, especially in individuals with IFI, which are mostly single-site, but there are also cases of two or even multi-site disseminated infections (Hou et al., 2010; Lahmer et al., 2022). In critically ill individuals with hepatic insufficiency and IFI, the most frequent site of infection is the lung (37.0%–56.0%); other sites are the gastrointestinal tract (1.1%–20.2%), urinary tract (4.3%–15.9%), abdominal cavity (2.9%–14.4%) and bloodstream (0.7%–5.8%), and fungal infections of the thoracic cavity, biliary tract, and central nervous system are also seen (Fernández et al., 2018; Piano et al., 2019; Libera et al., 2023). When the liver is not functioning properly, it can affect the metabolism and excretion of VRZ; this may impact the drug’s absorption and clearance rates from the body (Baririan et al., 2007; Verbeeck, 2008). Their Ctrough levels are significantly higher when they have hepatic insufficiency, which raises the risk of ADRs and can have a detrimental effect on their prognosis (Alffenaar et al., 2009; Ueda et al., 2009).

The most common pathogenic fungi of hepatic insufficiency combined with IFI are *Candida* spp. and Aspergillus spp. *Candida* spp. are mainly *Candida* albicans, accounting for more than 50%, and are the main pathogens of the intestinal tract, bloodstream, abdominal cavity, and urinary tract (Jenks et al., 2020; Silva et al., 2021; Hall et al., 2023). A multicenter study in Europe showed that fungal bloodstream infections were dominated by *Candida* albicans (54.4%), followed by *Candida* smooth (14.5%), *Candida* subsmooth (14.1%), *Candida tropicalis* (5.8%), *Candida* graminearum (2.5%), of which 34.9% of individuals suffered from septic shock (Bassetti et al., 2017; Medeiros et al., 2019). *Candida* albicans was also the main causative agent of fungal peritonitis (48.0%–81.8%), followed by *Candida klebsiella* (15.0%–25.0%), *Candida* smoothes (6.6%–20.0%), and novel Cryptococcus spp. were seen in some individuals (Tariq et al., 2019; Feldman et al., 2023). Pulmonary IFI is mainly caused by Aspergillus spp., with Aspergillus fumigatus being the most common, followed by Aspergillus flavus and Aspergillus niger; Aspergillus pyogenes and Aspergillus terreus are less frequently reported (Su et al., 2010; Jović et al., 2019; Lahmer et al., 2019). Cases of severe hepatic insufficiency combined with Pneumocystis pneumonia have also been reported to occur (Hadfield et al., 2019). In severe hepatic insufficiency, both natural and acquired immunity are severely impaired, resulting in decreased immunity, and often accompanied by intestinal dysfunction, intestinal mucosal edema, increased permeability, impaired intestinal barrier leading to flora translocation, intestinal microorganisms can enter the portal vein through the intestinal wall, coupled with serious damage to the liver mononuclear macrophage system, the ability to remove microorganisms is reduced, resulting in infection with bacteria, viruses and fungi and other pathogens the risk of infection with pathogens such as bacteria, viruses and fungi is significantly increased (Matsubara et al., 2016; Andrade et al., 2022). Therefore, individuals with hepatic insufficiency are more susceptible to developing IFI, and IFI usually occurs in the blood circulation and eventually causes systemic fungal infections, leading to conditions such as organ failure, sepsis, and fatal multi-organ dysfunction syndrome, for which VRZ is the first-line drug (Xing et al., 2017).

Severe hepatic insufficiency combined with IFI has an inadequate prognosis, with an upper morbidity and death rate, the clinical manifestations can be atypical, and diagnosing and treating it can be difficult. Antifungal drugs are mostly metabolized in the liver, which can cause highly toxic side effects (Cheong et al., 2009). According to relevant literature, individuals infected with *Candida* have a 30%–40% morbidity and mortality rate, while individuals infected with Aspergillus have an even higher rate of 50%–100% (Lahmer et al., 2016). *Candida* infection, invasive Aspergillus infection individuals to increase morbidity and mortality rate (Hwang et al., 2014). During liver transplantation, individuals who receive a new liver have an increased chance of getting fungal infections during and after the procedure (Kang et al., 2020). In recent years, prophylactic use of antifungal medications has helped to bring the overall prevalence of these infections down to 4%–8% (Lum et al., 2020; Khalid et al., 2021).

## 4 The impact of gene polymorphisms on VRZ

### 4.1 The impact of CYP2C19 on VRZ dose adjustment

VRZ is mainly metabolized in the liver and mediated by cytochrome P450 (CYP) 2C19 enzyme (Theuretzbacher et al., 2006). The CYP2C19 gene has genetic polymorphisms that can affect the pharmacokinetic characteristics of VRZ; in fact, these polymorphisms are responsible for 50% of the variability in VRZ. (Amsden and Gubbins, 2017). The gene that codes for CYP2C19 has more than 34 different versions, known as alleles; one of these alleles, called CYP2C19*17, has a mutation in the gene’s promoter region, making it more active than usual (Lee et al., 2022). It was found that mutant genes such as CYP2C19*2 and CYP2C19*3 were connected to pharmacokinetic parameters of VRZ, and individuals carrying mutant genes such as CYP2C19*2 and CYP2C19*3 had a slower clearance of VRZ, higher drug exposure, and greater fluctuations in drug concentration at the same dose (Hamadeh et al., 2017). It has been found that individualized adjustment of VRZ dose according to individuals’ CYP2C19 genotypes can reduce drug exposure and decrease the incidence of ADRs while ensuring drug efficacy (Amsden and Gubbins, 2017; Zhang et al., 2021). Individualized dose adjustment of VRZ may be necessary for different CYP2C19 genotypes. The proper dosage can be determined using TDM in conjunction with pharmacogenetic testing (He et al., 2020; Li et al., 2021).

The Clinical Pharmacogenetics Implementation Consortium (CPIC) categorizes individuals into five groups based on their genotype for CYP2C19 (Moriyama et al., 2017). These groups include CYP2C19 ultrarapid metabolizers (UMs), CYP2C19 rapid metabolizers (RMs), CYP2C19 normal metabolizers (NMs), CYP2C19 intermediate metabolizers (IMs), and CYP2C19 poor metabolizers (PMs) (Lee et al., 2022) (See Table 1: CYP2C19 phenotype classification). The differences in CYP2C19 genes between individuals can greatly affect how they respond to VRZ medication, particularly for those with liver problems. Studies have shown that identifying a patient’s CYP2C19 gene phenotype is essential in determining the appropriate VRZ dosage, as it can vary greatly from UMs to PMs (Ren et al., 2019). The impact of CYP2C19 is noted in the VRZ medication label approved by the FDA. However, there are currently no genetic variant-based dosing instructions available. To avoid potential problems, CPIC suggests utilizing antifungal medications that do not rely on the CYP2C19 enzyme metabolism for individuals with PMs/Ums, while standard VRZ dosing is recommended for other phenotypes, and the Dutch Pharmacogenetics Working Group (DPWG) recommends dose adjustment for both PMs and UMs phenotypes (García-García and Borobia, 2021; Maertens et al., 2021). The inconsistency and ambiguity of these guidelines may hinder clinicians’ practical application of these drugs.

**TABLE 1:** CYP2C19 phenotype classification.

Phenotype	Genotype	Effects on ctrough	Recommendations for adjustment from the CPIC
UMs (2–5%)	An individual with 2 increased function alleles (*17/*17)	The probability of attainment of therapeutic voriconazole Ctrough is small with standard dosing	Consider using an alternative agent, such as isavuconazole, liposomal amphotericin B, or posaconazole, as the primary therapy instead of voriconazole. These agents are not dependent on CYP2C19 metabolism
RMs (2–30%)	An individual with one common function allele and one increased function allele (*1/*17)	The probability of attainment of therapeutic voriconazole Ctrough is small with standard dosing	Consider using an alternative agent, such as isavuconazole, liposomal amphotericin B, or posaconazole, as the primary therapy instead of voriconazole. These agents are not dependent on CYP2C19 metabolism
NMs (35–50%)	An individual with 2 common function alleles (*1/*1)	Normal voriconazole metabolism	Start treatment with the recommended standard dosage
IMs (18–45%)	An individual with one common function allele and one no function allele or one no function allele and one increased function allele (*1/*2, *1/*3, *2/*17)	Higher dose-adjusted Ctrough of voriconazole compared with NMs	Start treatment with the recommended standard dosage
PMs (2–15%)	An individual with 2 no function alleles (*2/*2, *2/*3, *3/*3)	Higher dose-adjusted Ctrough of VRZ and may increase probability of adverse events	Consider using an alternative agent, such as isavuconazole, liposomal amphotericin B, or posaconazole, as the primary therapy instead of voriconazole. These agents are not dependent on CYP2C19 metabolism

UMs, CYP2C19 ultra-rapid metabolizers; RMs, CYP2C19 rapid metabolizers; NMs, CYP2C19 normal metabolizers; IMs, CYP2C19 intermediate metabolizers; PMs, CYP2C19 poor metabolizers.

The CYP2C19*1/*17 and *17/*17 genotypes conferred higher enzymatic activity to the RMs and UMs phenotypes, respectively, compared to NMs (Sim et al., 2006). The *2 and *3 alleles were loss-of-function variations. IMs with one such variant had significantly lower enzyme activity compared to NMs. However, PMs with two such variants showed no enzyme activity. According to Hamadeh et al. (2017), CYP2C19 genotype significantly impacts the risk of VRZ underexposure, individuals with *17/*17 genotypes (UMs) and around 50% of those with *1/*17 genotypes (RMs) were unable to achieve a therapeutic Ctrough (2–6 mg/L) when VRZ was administered based on body weight. UMs showed a decrease in VRZ Ctrough, resulting in a delay in reaching the target Ctrough; on the other hand, PMs exhibited an increase in Ctrough, which puts them at a higher risk of ADRs (Walsh et al., 2018). Hence, compared to NMs, UMs and RMs may require an increase in the VRZ dosage, while PMs may necessitate a reduction in the VRZ dosage (Lamoureux et al., 2016) (See Table. 2: Recommendations for dose adjustment for different CYP2C19 phenotypes). It is important to be aware that medications like omeprazole and cimetidine, which inhibit CYP2C19, can increase Ctrough levels in VRZ; conversely, taking certain CYP450 enzyme inducers at the same time can cause Ctrough levels to drop below the necessary therapeutic levels, resulting in clinical failure (Mikus et al., 2006). Existing meta-analyses indicate that PMs taking VRZ are at an upper risk of experiencing ADRs compared to NMs and IMs; however, other meta-analyses have not found a significant correlation between the two (Li et al., 2016; Amsden and Gubbins, 2017). Therefore, we still require extensive, high-quality trials to confirm these findings.

**TABLE 2:** Recommendations for dose adjustment for different CYP2C19 phenotypes.

First author year	Study design	Sample size	Phenotype	Recommendations for dose adjustment
Zubiaur et al. (2021)	prospective observational study	106	UMs	3 times the standard dose
			RMs	2 times the standard dose
			NMs	the standard dose
			IMs	0.5 times the standard dose
			PMs	0.25 times the standard dose
Tanaka et al. (2020)	prospective observational study	19	IMs	Reduce the initial maintenance dose
			PMs	
Blanco-Dorado et al. (2020)	prospective observational study	78	RMs	Increase the initial maintenance dose
			UMs	
Li et al. (2020)	prospective observational study	93	RMs	PO 400 mg, twice a day
			NMs	PO 400 mg, twice a day
			IMs	PO 200 mg, twice a day
Hicks et al. (2020)	prospective observational study	202	UMs	VRZ is recommended to be avoided
			RMs	PO 300 mg, twice a day
			NMs, IMs, PMs	PO 200 mg, twice a day
Miao et al. (2019)	retrospective cohort study	105	NMs	the standard dose
			IMs	1.64 times the standard dose
			PMs	2.61 times the standard dose
Lin et al. (2018)	prospective observational study	105	RMs	IV 300 mg, twice a day
			IMs	IV 200 mg/Oral 350 mg, twice a day
			PMs	IV 150 mg/Oral 250 mg, twice a day
PharmGKB (2017)	NA	NA	UMs	1.5 times the standard dose
			IMs	the standard dose
			PMs	0.5 times the standard dose
Lamoureux et al. (2016)	retrospective study	35	UMs	IV 6.75 mg/kg, twice a day
			RMs	IV 3.94 mg/kg, twice a day
			NMs	IV 2.57 mg/kg, twice a day
Wang et al. (2014b)	prospective observational study	144	PMs	PO 200 mg, twice a day
			non-PMs	IV 200 mg/PO 300 mg, twice a day

UMs, CYP2C19 ultra-rapid metabolizers; RMs, CYP2C19 rapid metabolizers; NMs, CYP2C19 normal metabolizers; IMs, CYP2C19 intermediate metabolizers; PMs, CYP2C19 poor metabolizers; PO, oral administration; IV, intravenous injection; NA, not applicable.

Even though many studies have shown how the CYP2C19 gene polymorphisms affect VRZ dosage adjustment, specific details and controversies still exist. PMs/IMs lead to elevated VRZ blood levels that may result in toxicity, such as hepatotoxicity or neurotoxicity, but the link between PMs/IMs and hepatotoxicity has not been established (Wang et al., 2014b). Additional investigation is necessary to fully comprehend the effects of CYP2C19*2 and CYP2C19*3 mutations on VRZ therapy response and hepatotoxicity. Differences in CYP2C19 genotype distribution in different populations may affect the applicability of dose adjustment strategies. For example, some studies have found a higher frequency of mutant phenotypes such as CYP2C19*2 in Asian populations, while enhanced phenotypes such as CYP2C19*17 predominate in European and American people, which may affect the accuracy and effectiveness of dose adjustment strategies (Mikus et al., 2011; Lee et al., 2021). Various studies indicate that the impact of CYP2C19 gene variations on the efficiency and security of VRZ may depend on the particular approach used for adjusting the dosage. For example, it has been suggested that individualized dose adjustment strategies may improve the efficacy and safety of VRZ more than conventional dose adjustment strategies in CYP2C19*2 and other mutant carriers (Hamada et al., 2013). Although CYP2C19 gene polymorphisms have an impact on the pharmacokinetic parameters and efficacy of VRZ, other causes, including individuals’ liver and kidney function and drug interactions, need to be considered in actual clinical application. Special attention should be given to the impact of drug-induced enzyme reactions on the alteration of related drug plasma concentrations, particularly when co-administered with hepatic enzyme inducers or inhibitors, to avoid ADRs (Hakkola et al., 2020) (See Table 3: Inhibitors and inducers of CYP2C19, CYP3A4, and CYP2C9). Therefore, the dose adjustment strategy should consider various factors rather than being based solely on CYP2C19 genotype (Moriyama et al., 2017).

**TABLE 3:** Inhibitors and inducers of CYP2C19, CYP3A4, and CYP2C9.

Liver enzymes	Inhibitors	Inducers
CYP2C19	Esomeprazole, omeprazole, fluconazole, voriconazole, chloramphenicol, artemisinin, isoniazid, fluoxetine hydrochloride, indomethacin, valproate sodium, oxcarbazepine, fluvastatin, lovastatin, nicardipine, amiodarone, zafirlukast, oral contraceptives, etc	Rifampicin, ritonavir, dexamethasone, Ginkgo biloba preparation, etc
CYP2C9	Amiodarone, nifedipine, nicardipine, fenofibrate, fluvastatin, tamoxifen, cimetidine, fluoxetine, paroxetine, sertraline, fluvoxamine, isoniazid, ketoconazole, fluconazole, voriconazole, sulfamethoxazole, Leflunomide, sodium valproate, zafirlukast, fluorouracil, etc	Barbiturates, bosentan, carbamazepine, rifampicin, dexamethasone, ritonavir, etc
CYP3A4	Amiodarone, verapamil, cimetidine, doxycycline, enoxacin, Ciprofloxacin hydrochloride, erythromycin, clarithromycin, ketoconazole, miconazole, fluconazole, itraconazole, voriconazole, ritonavir, etc	Glucocorticoids, phenobarbital, phenytoin sodium, carbamazepine, oxcarbazepine, topiramate, rifampicin, pioglitazone, etc

### 4.2 The impact of CYP3A on VRZ

CYP3A is the most prevalent metabolic enzyme in the liver and is engaged in the metabolism of 45%–60% of frequently used medications; CYP3A4 and CYP3A5 are the most significant drug-metabolizing enzymes in this regard (Wojnowski, 2004). CYP3A5 accounts for approximately 17%–60% of hepatic CYP3A and has a similar substrate specificity to CYP3A4; however, even with the same substrate, CYP3A4 exerts a higher metabolic efficiency (Klyushova et al., 2022). CYP3A4 is the main metabolic enzyme for VRZ hydroxylation metabolism, while CYP3A5 plays a relatively weak role in VRZ hydroxylation metabolism, and studies have shown that the hydroxylation metabolism of VRZ by CYP3A4 and CYP3A5 is relatively enhanced when CYP2C19 enzyme activity is diminished (Murayama et al., 2007). The CYP3A4 gene is a key enzyme in VRZ metabolism, and its genotype is associated with the pharmacokinetic and pharmacodynamic properties of VRZ. Still, compared with CYP2C19, CYP3A4 affects VRZ metabolism *in vivo* to a lesser extent, approximately 1/50th of CYP2C19(Hyland et al., 2003). Although CYP3A4 has been addressed in several previous studies, no genotypes explained the phenotype until two SNPs, rs4646437 and rs35599367, were found to be associated with the Ctrough of VRZ. The research findings reveal that the rs4646437 polymorphism significantly influences the mean blood drug concentration of VRZ, with the T variant allele being associated with higher blood drug concentrations (Gautier-Veyret et al., 2015; He et al., 2015). Walsh et al. found that polymorphisms such as CYP3A4*22 and CYP3A4*23 may impact the metabolism of VRZ, and CYP3A4 *22 was associated with higher VRZ concentrations compared to CYP3A4 *1/*1 (Walsh et al., 2018). Meanwhile, some studies indicate that genetic variations of CYP3A4 and CYP3A5 have little impact on the pharmacokinetics of VRZ (Lee et al., 2012; Chuwongwattana et al., 2020). Diverse studies’ findings on how CYP3A4 genotype affects VRZ are equivocal; more research is required to determine how the two are related. Research on how CYP3A5 affects the pharmacokinetics of VRZ has also produced inconsistent results. According to Weiss et al., there is no apparent connection between CYP3A5*3 mutations and VRZ pharmacokinetics (Weiss et al., 2009). According to Levin et al. (2007), the amount of hepatic drug-metabolizing enzymes in the blood may indicate high levels of VRZ plasma concentration-induced liver toxicity; the study also discovered that the liver damage was not related to the CYP3A5*3 allele. However, a study conducted in a laboratory setting has demonstrated that individuals with the genetic variant CYP3A5*3/*3 experience a threefold increase in AUC when taking VRZ compared to those with at least one functional allele (Yamazaki et al., 2010).

### 4.3 The impact of CYP2C9 on VRZ

Studies have revealed that CYP2C19 is the primary enzyme responsible for the nitrogen-based oxidative metabolism of VRZ; however, CYP2C9 can also contribute to this process to a lesser extent (Dorji et al., 2019). Lee et al. (2002) showed that there is a link between the variability of VRZ blood concentration and the CYP2C9*2 and CYP2C9*3 alleles. The CYP2C9*13 allele is the first novel variant of CYP2C9 identified in Chinese and is important in determining the metabolic capacity of CYP2C9. Some studies indicate that the CYP2C9*13 gene variation can decrease drug clearance from the bloodstream (Si et al., 2004; Zhang et al., 2007). There are few conclusions regarding clinical aspects supporting the impact of different genotypes of CYP2C9 on VRZ metabolism, and there are some indications that CYP2C9 genotypes may not be associated with VRZ pharmacokinetics. The current research has made the function of CYP2C9 in VRZ metabolism somewhat controversial. According to Niwa et al., the CYP2C9 *2 allele results in less effective inhibition of CYP2C9 by VRZ compared to the CYP2C9 *1 and CYP2C9 *3 alleles (Niwa and Hata, 2016). It has been reported that the pharmacokinetic parameters of VRZ were not altered in individuals genotyped as CYP2C9 *2/*2 pure siblings (Liu and Mould, 2014). Furthermore, a study conducted on 35 healthy participants revealed that there was no impact of CYP2C9 on VRZ pharmacokinetic parameters, as determined by a multiple regression analysis of VRZ pharmacokinetics (Weiss et al., 2009). In conclusion, there are varying results from different studies regarding the impact of CYP2C9 genotype on VRZ, further research is necessary to reach a definitive conclusion.

### 4.4 The impact of ABCB1 on VRZ

P-glycoprotein (ABCB1) is one of the crucial transporter proteins in the human body and is essential for maintaining biological barriers (Tulsyan et al., 2016). Currently, there have been over 50 SNPs documented in the ABCB1 gene (Tanabe et al., 2001). According to Cascorbi et al., a specific variation, rs1045642, in exon 26 of the ABCB1 gene can lead to a decrease in protein function (Cascorbi et al., 2001). ABCB1 also has genetic polymorphisms that affect its transport activity and, thus, the pharmacokinetic parameters of its transported substrates (Sauna et al., 2007). It was found that VRZ can interact with CaMdr1p, a homolog of yeast P-glycoprotein and that VRZ can mildly inhibit the activity of P-glycoprotein, corroborating that VRZ may be a substrate of ABCB1(Wakieć et al., 2007). Few studies have been conducted on the effects of ABCB1 gene polymorphisms on VRZ metabolism, and no clear conclusions have been obtained. One study found that ABCB1 gene polymorphism has an impact on VRZ clearance (Weiss et al., 2009). It has been shown that the AA allele of the rs1045642 polymorphic locus carrying the ABCB1 gene is associated with reduced VRZ metabolism in healthy individuals compared to the GG genotype (Weiss et al., 2009; Allegra et al., 2018). However, Recent studies have shown that the ABCB1 gene polymorphism does not significantly impact blood concentrations of VRZ (Chuwongwattana et al., 2020). Therefore, To fully understand the impact of ABCB1 gene variations on VRZ metabolism, it is necessary to conduct studies using larger sample sizes encompassing different races.

### 4.5 The impact of other gene polymorphisms on VRZ

In addition to the genetic involvement of CYP2C19, CYP3A4, CYP2C9, and ABCB1, several other genetic variants may also broadly affect VRZ concentrations in individuals. According to the study, subjects carrying the rs3781727 variant of the SLCO2B1 gene had reduced and delayed oral absorption of VRZ, and genotype CC + CT was associated with reduced VRZ exposure in healthy individuals compared to genotype TT (Lee et al., 2020). The presence of the AA genotype at the rs2461817 polymorphic site in the NR1I2 gene is associated with a decrease in VRZ concentrations; furthermore, the presence of the GG allele at the rs6785049 polymorphic site and the CC allele at the rs3814057 polymorphic site in the NR1I2 gene, the AA allele at the rs2266780 polymorphic site in the FMO3 gene, and the AA allele at the rs2266780 polymorphic site in the POR gene, as well as the GG allele at the rs10954732 polymorphic site in the POR gene, is correlated with an increase in VRZ concentrations (Zeng et al., 2020). Regression analysis confirmed the potential function of the rs4149117 GT/TT genotype group of the SLCO1B3 gene in predicting Ctrough reduction by VRZ, and one study showed that individuals carrying the GT + TT allele of the rs4149117 polymorphic locus of the SLCO1B3 gene were associated with reduced Ctrough in children (Allegra et al., 2018). The ABCC2 gene encodes a transporter protein that has a tremendous impact on the transport and clearance of VRZ, and the rs717620 polymorphic locus CT + TT allele carrying the ABCC2 gene and the rs13120400 polymorphic locus CC allele carrying the ABCG2 gene were also associated with elevated Ctrough in children (Allegra et al., 2018).

Overall, among the genetic influences on VRZ dose adjustment, CYP2C19 gene polymorphisms were the most influential, accounting for approximately 50% of VRZ variability (Amsden and Gubbins, 2017). Although CYP3A4 is associated with the pharmacokinetic and pharmacodynamic properties of VRZ, the effect of CYP3A4 on the metabolism of VRZ *in vivo* is less than that of CYP2C19, which is about 1/50 of CYP2C19 (Hyland et al., 2003; Murayama et al., 2007). The effect of CYP2C9 on the dose adjustment of VRZ is more slight, and it is only involved in a small part of the nitrogen oxidation metabolism of VRZ (Dorji et al., 2019). Although several studies have shown that CYP3A5, ABCB1, SLCO2B1, NR1I2, FMO3 and other genes have an effect on VRZ metabolism, these effects are relatively small compared with CYP2C19, CYP3A4, and CYP2C9. Moreover, there is a considerable amount of confounding factors and a lack of consistent conclusions in these studies, warranting further research in this area.

## 5 The recommended dose of VRZ in individuals with IFI

The recommended dose of VRZ for treating IFI in adults varies between countries and regions. Thus, it is important to adjust the dosage for each individual (See Table 4: Comparison of recommended doses in different countries). Overall, the recommended doses varied somewhat between countries. Still, all had the same intravenous loading dose and maintenance dose, and there was less variation between countries in oral dosing, and all recommended inter-individual dose adjustments based on Ctrough.

**TABLE 4:** Comparison of recommended doses in different countries.

Country	Intravenous infusion		Oral administration		Recommendations for dose adjustment
	Loading dose	Maintenance dose	Loading dose	Maintenance dose	
China	6 mg/kg every 12 h	4 mg/kg every 12 h	weighing more than 40 kg: 400 mg every 12 h; weighing less than 40 kg: 200 mg every 12 h	weighing more than 40 kg: 200 mg every 12 h; weighing less than 40 kg: 100 mg every 12 h	The dosage should be modified for each patient based on weight, disease features, drug metabolism, liver, kidney, and Ctrough (Chen et al., 2018)
United States	6 mg/kg every 12 h	4 mg/kg every 12 h	6 mg/kg every 12 h (Intravenous infusion)	weighing more than 40 kg: 200 mg every 12 h; weighing less than 40 kg: 100/150 mg every 12 h	The prescribed dose interval should be modified according to the patient’s medication metabolism, liver function, and renal function (Patterson et al., 2016)
EU	6 mg/kg every 12 h	4 mg/kg twice daily	weighing more than 40 kg: 400 mg every 12 h; weighing less than 40 kg: 200 mg every 12 h	Weighing more than 40 kg: 200 mg twice daily; weighing less than 40 kg: 100 mg twice daily	Individuals with compromised liver function and drug interactions should have their dosages customized based on their Ctrough levels (Ullmann et al., 2018)
United Kingdom	6 mg/kg every 12 h	4 mg/kg twice daily	weighing more than 40 kg: 400 mg every 12 h; weighing less than 40 kg: 200 mg every 12 h	weighing more than 40 kg: 200 mg twice daily; weighing less than 40 kg: 100 mg twice daily	The dosage should be adjusted accordingly, taking into consideration the patient’s drug metabolism, potential drug interactions, as well as liver and kidney function (Ashbee et al., 2014)
Japan	6 mg/kg every 12 h	4 mg/kg twice daily	weighing more than 40 kg: 300 mg twice daily (For the first 2 days). weighing less than 40 kg: 150 mg twice daily (For the first 2 days)	weighing more than 40 kg: 150/200 mg twice daily; weighing less than 40 kg: 100 mg twice daily	Ctrough monitoring is advised, along with tailored dosage and dosing interval adjustments for VRZ based on genetic polymorphism and drug metabolism (Roberts et al., 2012)

Different countries have varying Ctrough levels. For instance, Chinese guidelines suggest a minimum of 0.5 mg/L and a maximum of 5 mg/L for VRZ target Ctrough (Chen et al., 2018). The Japanese guidelines state that Ctrough ≥12 mg/L can achieve clinical efficacy, and individuals with Ctrough >4–5 mg/L should be monitored for elevated related indicators (Roberts et al., 2012). According to the 2016 guidelines in the US, individuals should maintain a minimum requirement of 1–1.5 mg/L and a maximum requirement of 5–6 mg/L for Ctrough (Patterson et al., 2016). European 2017 guidelines recommend that the lower limit of Ctrough in individuals should be 1–1.5 mg/L, and the recommended Ctrough for severe infections is 2–6 mg/L (Ullmann et al., 2018). The British Society for Medical Mycology (BSMM) antifungal drug TDM guideline recommendation defines the VRZ treatment window as 2 ∼ 6 mg/L (Ashbee et al., 2014).

In terms of dose adjustment, the “VRZ Personalized Medication Guidelines,” published by the Chinese Pharmacology Society, recommend using a population pharmacokinetic model based on the Chinese public to adjust VRZ dosing. For individuals with a steady-state Ctrough below the lower limit of the target Ctrough or poor efficacy, it is recommended that the VRZ maintenance dose be increased by 50% and then adjusted according to Ctrough; for individuals with a steady-state Ctrough above the upper limit of the target Ctrough and below 10 mg/L, and in the absence of grade 2 or higher adverse events, it is recommended that the VRZ maintenance dose be diminished by 20% and then adjusted according to Ctrough; For individuals with steady-state Ctrough above 10 mg/L or Grade 2 adverse events, then VRZ is recommended to be discontinued for one dose, followed by a maintenance dose reduction of 50%, followed by adjustment based on Ctrough (Chen et al., 2018).

## 6 VRZ dose adjustment in individuals with hepatic insufficiency

Individuals with hepatic insufficiency may face a higher risk of ADRs due to the potential accumulation of VRZ caused by decreased hepatic blood flow and enzyme activity. Individuals with hepatic insufficiency are advised to follow the VRZ instructions. For those with mild to moderate hepatic insufficiency (Child-Pugh A/B), a standard loading dose and a maintenance dose that is half the usual dosage are recommended (Chen and Chen, 2021). However, it is unclear what the proper dosing of VRZ should be for individuals with serious hepatic insufficiency (Child-Pugh C). Studies have indicated that the recommended VRZ loading dose and maintenance dose halving are not suitable and that reducing the maintenance dose by half can result in perilously high drug levels in these individuals (Wang et al., 2018b; Spernovasilis and Kofteridis, 2018). Therefore, conducting a pharmacokinetic study of VRZ in this particular population is crucial to develop suitable dosage schedules (See Table 5: Recommendations for dose adjustment in hepatic insufficiency).

**TABLE 5:** Recommendations for dose adjustment in hepatic insufficiency.

First author year	Study design	Sample size	Liver function grading	Recommendations for dose adjustment
Cai et al. (2023)	retrospective study	308	Child-Pugh C	Loading dose: no recommendation Maintenance dose: 200 mg every 24 h
Lin et al. (2022)	prospective observational study	26	Child-Pugh A/B	Loading dose:5 mg/kg every 12 h
				Maintenance dose: 100 mg every 12 h/200 mg every 24 h
			Child-Pugh C	Loading dose:5 mg/kg every 12 h
				Maintenance dose: 50 mg every 12 h/100 mg every 24 h
Zhao et al. (2021)	prospective observational study	43	Child-Pugh C	Loading dose:200 mg every 24 h
				Maintenance dose: 100 mg every 24 h
Wang et al. (2021)	Retrospective study	120	Child-Pugh A/B	Loading dose: 200 mg every 12 h
				Maintenance dose: 75 mg every 12 h/150 mg every 24 h
			Child-Pugh C	Loading dose:200 mg every 12 h
				Maintenance dose: 50 mg every 12 h/100 mg every 24 h
Tang et al. (2021)	prospective observational study	51	TBIL-1	Loading dose:200 mg every 12 h
				Maintenance dose: 100 mg every 12 h
			TBIL-2	Loading dose:200 mg every 12 h
				Maintenance dose: 50 mg every 12 h/100 mg every 24 h
			TBIL-3	Loading dose: 200 mg every 12 h
				Maintenance dose: 50 mg every 24 h
Ren et al. (2019)	retrospective study	180	Child-Pugh A/B	Loading dose: no recommendation Maintenance dose: 75 mg every 12 h
			Child-Pugh C	Loading dose: no recommendation
				Maintenance dose: 100 mg every 24 h
Zhao et al. (2019)	retrospective study	117	Child-Pugh C	Loading dose: no recommendation
				Maintenance dose: 100 mg every 12 h
Yamada et al. (2018)	retrospective study	6	Child-Pugh C	Loading dose: no recommendation Maintenance dose: 100–130 mg every 24 h
Wang et al. (2018b)	Retrospective Study	78	Child-Pugh B/C	The recommended maintenance dose (200 mg every 12 h) and halved maintenance dose (100 mg every 12 h) result in high Ctrough
Wang et al. (2018a)	Retrospective Study	34	Child-Pugh C	Maintenance doses (100 mg every 12 h/200 mg every 24 h) result in high Ctrough
Gao et al. (2018)	retrospective study	20	Acute Chronic Liver Failure	Loading dose: 200 mg every 12 h
				Maintenance dose: 100 mg every 24 h
Liu et al. (2017)	case report	1	Child-Pugh C	Loading dose: no recommendation
				Maintenance dose: 100 mg every 24 h

TBIL-1, TBIL <51 μmol/L; TBIL-2, 51 μmol/L ≤ TBIL <171 μmol/L; TBIL-3, TBIL ≥171 μmol/L.

The 12 studies have examined the use of VRZ in individuals with hepatic insufficiency. All have concluded that the currently recommended dose is unsuitable for these individuals and requires adjustment. Of these, 10 studies provided specific recommendations for dose adjustments, but only 5 gave both loading and maintenance doses, while the other 5 only provided maintenance doses. 8 retrospective multisample studies and 1 case report have shown that the standard loading dose and maintenance dose for individuals with hepatic insufficiency may not be appropriate, particularly for those in Child-Pugh class B and C. This is because of their higher Ctrough levels, which increase the risk of serious ADRs. To prevent elevated Ctrough levels and associated ADRs, it is advisable to consider lower doses, longer dosing intervals, and early TDM for these patients.

In individuals with hepatic dysfunction, total bilirubin has been identified as a crucial factor in predicting the pharmacokinetic parameters of VRZ. Optimizing the VRZ dosage to align with the total bilirubin levels can enhance treatment effectiveness. A prospective observational study categorized individuals with hepatic insufficiency into three levels based on total bilirubin levels and determined the optimal therapeutic dosage of VRZ for each bilirubin level (refer to Table 5); additionally, the study found that the pharmacokinetics of VRZ can be appropriately described using a one-compartment model with first-order absorption and elimination in individuals with hepatic dysfunction (Tang et al., 2021). These findings align with former retrospective studies and the research conducted by Pascual et al. (2012) and Wang et al. (2014a) on the pharmacokinetics of VRZ in individuals. A population-based pharmacokinetic modeling study showed that individuals with Ctrough >5.12 mg/L were more likely to experience VRZ-related ADRs, and individuals with hepatic insufficiency should receive a reduced half-load dose regimen compared with individuals with normal liver function, and the VRZ maintenance dose should be reduced to one-third for Child-Pugh A/B individuals and one-quarter for Child-Pugh C individuals (Wang et al., 2021). CYP2C19 phenotype plays a crucial role in selecting VRZ treatment regimens in individuals with liver insufficiency. When CYP2C19 activity is reduced, individuals with the same degree of liver insufficiency can further reduce the dose of VRZ. The results of a dosing regimen optimization based on MonteCarlo simulation showed that the maintenance dose of VRZ should be decreased to less than 50% in individuals with mild to moderate hepatic insufficiency with extensive CYP2C19 metabolism and o 1/4 in individuals with moderate to severe hepatic insufficiency (Ren et al., 2019). Dote et al. found that taking glucocorticoids alongside VRZ lowers plasma levels, while taking proton pump inhibitors increases plasma levels (Dote et al., 2016). Some studies have indicated that steroids are a hazard element for fungal infections in individuals with liver failure; Liu et al. (2017) found that VRZ is safe in individuals with fungal pneumonia and that low-maintenance doses of VRZ (100 mg/d) can achieve effective Ctrough without causing liver damage, but Ctrough of VRZ should be carefully monitored. A prospective observational study has shown that the regular VRZ dose can be increased by 50 mg in individuals with hepatic insufficiency at a MIC of 1 mg/L, but Ctrough needs to be monitored carefully to avoid severe ADRs; When the MIC is ≥ 2 mg/L, other alternative drugs are recommended, and depending on the type of fungal pathogen and its susceptibility to VRZ, lower doses or longer dosing intervals should be recommended to individuals with hepatic insufficiency (Lin et al., 2022).

## 7 Discussion

VRZ, a widely used broad-spectrum antifungal medication for treating fungal infections, shows significant variability in its pharmacokinetics and pharmacodynamics among individuals. This is due to the involvement of multiple metabolic pathways and influencing factors. More and more research has emphasized the significance of genetic polymorphisms and hepatic insufficiency in determining appropriate VRZ dosage adjustments for individuals with IFI. Recent studies have investigated the potential relationship between genetic variations, such as CYP2C19, CYP3A4, ABCB1, ABCC2, FMO3, and POR, and the pharmacokinetics and pharmacodynamics of VRZ. Among these genes, CYP2C19 has the strongest impact on VRZ metabolism and clearance, followed by CYP3A4. Certain variants, like CYP2C19*2 and CYP2C19*3, reduce the enzymatic activity of CYP2C19, which results in higher drug exposure. On the contrary, variants such as CYP2C19*17 enhance CYP2C19 activity, resulting in faster VRZ metabolism and reduced drug exposure. ABCB1 and ABCC2 genotypes may influence VRZ transport and distribution, while FMO3 and POR genotypes could potentially impact its metabolism and clearance. However, it is important to note that the findings from different studies are not always consistent, warranting further research to understand the specific effects of each genotype on VRZ.

The treatment of patients with hepatic insufficiency complicated by IFI is a clinical challenge and a topic of great interest. While existing pharmacokinetic studies, clinical trials, and post-marketing safety data of available antifungal agents can assist clinicians in optimizing antifungal treatment regimens in patients with mild to moderate hepatic insufficiency and IFI, the recommended dosage adjustments for patients with severe hepatic insufficiency remain unclear in most current guidelines. Furthermore, the majority of dose adjustment studies for VRZ in patients with hepatic insufficiency have primarily focused on maintenance doses, with limited recommendations for loading doses. Moreover, there are discrepancies in the recommended adjustment doses across different studies, highlighting the lack of consensus. Therefore, further pharmacokinetic and clinical research is warranted to guide the use of VRZ in patients with hepatic insufficiency. Additionally, TDM of antifungal agents should be strengthened in clinical practice for patients with hepatic insufficiency and IFI to prevent or promptly identify hepatic and renal impairment, thereby avoiding adverse clinical outcomes. Furthermore, there is limited evidence and research on the dose adjustment of antifungal agents based on genotype and phenotype in patients with hepatic insufficiency, necessitating more extensive investigation in this aspect.

The current recommendations in guidelines and package inserts regarding patients with mild to moderate hepatic insufficiency (Child-Pugh A and B) who are prescribed VRZ suggest standard loading doses and halved maintenance doses, but this approach is likely to result in high Ctrough levels in patients, making it potentially inappropriate. Several ADRs associated with VRZ use have been found to directly correlate with Ctrough levels (Zonios et al., 2008; Tang et al., 2021). A meta-analysis conducted to assess the utility of TDM revealed a significantly higher frequency of toxic adverse events in patients with Ctrough levels ranging from 4.0 to 6.0 mg/L compared to those with lower Ctrough levels (Luong et al., 2016). Furthermore, a review of plasma monitoring studies for VRZ demonstrated that maintaining a treatment window of >1–2 mg/L and <5–5.5 mg/L was associated with improved efficacy and reduced toxicity (Karthaus et al., 2015). Additionally, a randomized controlled trial evaluating the utility of TDM in patients receiving VRZ treatment for IFI found that patients undergoing TDM exhibited a significant increase in complete or partial treatment response, with fewer discontinuations due to adverse events (Park et al., 2012). Moreover, the “VRZ personalized dosing guideline” strongly recommends Ctrough monitoring for patients with hepatic insufficiency, those co-administering drugs that affect VRZ pharmacokinetics, patients with CYP2C19 gene mutations, patients experiencing VRZ-related adverse events or suboptimal treatment efficacy, and critically ill patients with life-threatening fungal infections (Chen et al., 2018). It is evident that conducting Ctrough monitoring in hepatic insufficiency patients using VRZ is highly necessary. TDM should be initiated early when administering VRZ, and if steady-state Ctrough falls below the lower limit or if treatment efficacy is suboptimal, dosage adjustments should be made according to the dose adjustment scheme outlined in the “VRZ personalized dosing guideline.” Additionally, the CYP2C19 gene phenotype plays a crucial role in determining VRZ dosage in patients with hepatic insufficiency. When making dosage adjustments, special attention should be given to the impact of CYP2C19 gene phenotype in hepatic insufficiency patients on VRZ dosage adjustments (Tang et al., 2019).

From an individualized dosing perspective, hepatic insufficiency and genetic polymorphisms are two important factors influencing the administration dosage of VRZ in patients. In terms of the genetic impact on VRZ dose adjustments, the majority of the influence is attributed to the involvement of CYP2C19, CYP3A4, and CYP2C9, with CYP2C19 being particularly significant (accounting for approximately 50% of VRZ variability). Therefore, it is crucial to focus on the impact of CYP2C19, CYP3A4, and CYP2C9 gene phenotypes in hepatic insufficiency patients on VRZ plasma concentrations, as this holds positive implications for the successful treatment of hepatic insufficiency with concomitant invasive fungal infections. Other genes such as CYP3A5, ABCB1, SLCO2B1, NR1I2, and FMO3, which have lesser impact, may also be considered to some extent. To validate the safety and efficacy of VRZ dose adjustment strategies based on genotypes and liver function, future research should further investigate how to optimize the therapeutic approach of VRZ and better utilize genetic testing and clinical practice guidelines to guide VRZ dosage adjustments. This includes expanding the sample size and enhancing comparative studies among different populations, which can further elucidate the influence of genetic polymorphisms on VRZ pharmacokinetics and pharmacodynamics. Furthermore, since hepatic insufficiency patients often present with other diseases and receive concurrent medication, these factors may also impact VRZ pharmacokinetics and dose adjustments. Lastly, further research is necessary to examine the influence of genetic polymorphisms on ADRs, in order to guide clinical drug use and personalized treatment.

In conclusion, pharmacogenomics-based VRZ dose adjustment offers accurate and personalized treatment for hepatic insufficiency, improving outcomes and reducing ADRs. Compared to those with normal liver function, patients with hepatic insufficiency require lower drug doses and longer dosing intervals. Early TDM is crucial to mitigate potential adverse events. Additionally, the impact of CYP2C19, CYP3A4, and CYP2C9 genes on hepatic insufficiency patients with IFI should be carefully considered. Future high-quality pharmacogenomics trials are urgently needed to enhance evidence-based medicine and pharmacology for the diagnosis and treatment of hepatic insufficiency patients with IFI.
